# The nature of science identity and its role as the driver of student choices

**DOI:** 10.1186/s40594-018-0140-5

**Published:** 2018-11-30

**Authors:** Paulette Vincent-Ruz, Christian D. Schunn

**Affiliations:** Learning Research and Development Center, University of Pittsburgh, Pittsburgh, USA

## Abstract

**Background:**

A major concern in science education involves the under-representation of many groups in science and technology fields, especially by gender (Brotman and Moore, J Res Sci Teach 45:971–1002, 2008; Clark Blickenstaff, Gend Educ 17:369–386, 2006), stemming from an intersection of systemic obstacles (Cantú, Equity Excell Educ 45:472–487, 2012; Rosa and Mensah, Phys Rev Phys Educ Res 12:020113, 2016). Research on persistence of minoritized populations within science trajectories has often highlighted identity as particularly important (Archer et al., Sci Educ 94:617–639, 2010; Barton and Calabrese, Am Educ Res J 50:37–75, 2007; Barton et al., Am Educ Res J 50:37–75, 2013; Merolla and Serpe, Soc Psychol Educ 16:575–597, 2013).

**Results:**

This study quantitatively investigated the nature of science identity in over 1300 seventh and ninth grade students from a range of urban US public schools using survey data on science identity, choice preferences, and optional science experiences. Factor analyses validated this conceptualization of science identity as integrating perceived internal and external identity components. Regression analyses revealed the importance of this conceptualization of science identity for driving students’ choices at this crucial developmental period. Furthermore, science identity had a complex differential function in supporting students’ optional science choices by gender.

**Conclusions:**

The novel contribution to the science identity field highlights the specific multi-component ways in which students endorse science identity in middle school and early high school. There was an important finding that science identity has a complex differential function in supporting student’s optional science choices by gender. Thus, at this age, developing a strong science identity is especially critical for girls.

**Electronic supplementary material:**

The online version of this article (10.1186/s40594-018-0140-5) contains supplementary material, which is available to authorized users.

## Background

A major concern in science education involves the under-representation of many groups in science and technology fields, especially by gender (Brotman and Moore 2008; Clark Blickenstaff 2006) and race/ethnicity (Archer et al. 2014; McGee and Bentley 2017). Particularly, there is the need to understand how major systems of oppression (e.g., racial, heteropatriarchal) hinder the development of science-related attitudes (Cantú 2012; Rosa and Mensah 2016). Among the attitudes connected with under-representation in science, identity has generally received less attention than other attitudinal constructs (e.g., in comparison with interest or self-efficacy), and it has been studied from highly varied disciplinary perspectives (e.g., science education, social psychology, educational psychology, and sociology) with strong conceptual and methodological differences. As a result, there are key open questions about the nature and measurement of science identity. The objective of this study was to quantitatively investigate the nature of science identity in middle-school and high-school students with a focus on what are the components of identity and whether this conceptualization of identity was useful for predicting participation in science at this crucial developmental period.

Identity can be defined as the composition of self-views that emerge from participation in certain activities and self-categorization in terms of membership in particular communities or roles (Stets and Burke 2000). More generally, when it comes to science identity, research has suggested that it not only involves whether an individual wants to become a “science type person,” but also as the socialization of individuals into the norms and discourse practices of science (Brown 2004). That is, identity is built from internalization from our experiences and socially constructed with others in a particular context.

### The nature of science identity

Researchers have presented three conceptualizations for what drives science identity: (1) *a sense of community and affiliation* (Carlone and Johnson 2007); (2) *built by consistent extrinsic and intrinsic attitudinal factors* (Aschbacher et al. 2010); (3) a *match between school science and real science* (Archer et al. 2010).

#### A sense of community and affiliation

Youth development can involve a tension between differentiation (how am I different?) and fitting-in (do I match group norms?), especially during adolescence (Kroger 2003). Many contextual factors shape these interactions, including stereotypes rooted in historical inequities (Schiebinger 2000). Further, understanding the role of science identity in persistence involves understanding how people negotiate the cultural norms within their community communities and in turn become affiliated with or alienated from science (Stets et al. 2017). The perceived interactions with others are likely critical in influencing identity development and internalization, particularly via perceptions of how others view them and how these views are built on these systemic inequities. Influential others (family, friends, and teachers/mentors) can play a large role in providing a feeling of community and affiliation, which then shapes identity, especially in early adolescence. An open question, though, is whether this perception of whether influential others view the individual defines their identity or simply acts as one of many attitudes, beliefs, and experiences that influence identity development overall.

#### Built by consistent extrinsic and intrinsic attitudinal factors

Several attitudinal constructs have been linked to science identity. Most commonly, interest (intrinsic motivation) has been linked as a primary driver of science identity: the bigger the science interest, the more solidified the science identity (Maltese and Tai 2010). Other conceptualizations assume that when interest leads to participation in science pathways and this participation leads to the development of career goals, then a science identity exists (Crowley et al. 2015). Furthermore, under the expectancy-value theory, science identity can lead to science-related choices when the learner also has strong perceptions about the (extrinsic) value of science and high levels of science self-efficacy or competency beliefs (Eccles et al. 2015).

However, it is important to note that there is disagreement whether the related attitudinal constructs drive identity development or whether these other attitudinal constructs are part of identity. For example, measures of identity often included items closely associated with these other concepts (Chang et al. 2016; Hazari et al. 2013; Trujillo and Tanner 2014). This raises an important question regarding the nature of science identity: is science identity different from these other attitudinal constructs in that students can be high or low on science identity independently of being high or low on these other attitudes?

#### Match between school science and real science

Learners may form a topical identity (e.g., science identity) by comparing their own performance/characteristics with the perceived characteristics of adults associated with the topic (e.g., scientists). The experiences youth have with science in school shape the perceptions they have about their performance/ characteristics. Unfortunately, many students perceive a mismatch between what it means to do science in the classroom and what science in real life entails (Zhai et al. 2013), and this mismatch influences identity development (Braund and Driver 2005; Emvalotis and Koutsianou 2017; Tan et al. 2015; Zhai et al. 2013).

More importantly, most science experiences at this age will come from formal environments rather than informal environments. Informal environments can provide minoritized students with specific opportunities to understand themselves as scientists and have a more realistic experience of how science works (Farland-Smith 2012). However, there are important concerns about access when it comes to informal environments (Dawson 2014) in the sense that there is not equal access by demographic variables as well as optional experiences producing positive feedback loops that accentuate initially small differences. Thus, it is important to understand the ways in which identity connects to participation in optional experiences (e.g., how robustly is it connected to participation across contexts?).

### Conceptualizing components of science identity

The literature is clear that influential others are likely to be important in the development of science identity for a variety of possible reasons. However, the literature on science identity is inconsistent conceptualizations regarding whether science identity is a latent construct built upon other attitudinal drivers such as interest and external perceptions (Hazari et al. 2013), an expected success from science experience (Barton and Tan 2010; Trujillo and Tanner 2014; Wigfield and Eccles 2000), or an independent construct. The literature is also inconsistent in methods regarding whether identity can only be assessed indirectly through the actions a learner takes (Archer et al. 2010; Barton et al. 2013) or whether it can be assessed through asking learners via surveys about their retrospective internalization of the identity (Barton, Kang & Tan 2013). We explore the survey approach and then apply common psychometric analysis techniques to examine the internal components of identity as well as its independence from other attitudinal constructs to improving understanding of what should be included in such a conceptualization. We also tested its predictive validity—constructs are useful when they organize phenomena. Such foundational construct testing and development work is important when a field of research has relatively high levels of disagreement about the nature of a construct and how it should be measured.

Construct development in a complex domain like science identity should involve a variety of methods, particularly a balance between qualitative and quantitative methods. There have already been many rich qualitative investigations of science identity, especially case studies constructed through semi-structured interviews. For example, Kozoll & Osborne, (2004) and (Polman & Miller, 2010) conducted interviews focused on how students’ experiences with science influenced their pathways, highlighting the role of identity. Similarly, Archer et al. (2010) connected students’ views of science to their aspirations for the future. However, it is not clear whether career aspirations should be taken as synonymous with identity since career aspirations can also be influenced by other external factors. Gee’s (2000) oft-cited definition of identity speaks of “who one wants to become,” but this definition is also unclear about when something becomes part of the self. This concern about current vs. career orientation in identity is especially an issue for younger learners who are still many years away from a professional role, and thus a science identity might exist without a commitment to a particular career.

By contrast, Aschbacher et al. (2010) focused more on peer and family expectations of science, arguing for the centrality of these perceived expectations on identity. However, it is unclear whether such expectations are part of an identity or whether they shape identity (Hazari et al. 2010). Clearly, the actual expectations that influential others (e.g., family, friends/peers, teachers/mentors) hold are external to the learner and therefore would be factors that shape identity rather than be a component of identity itself. Particularly, these external actors can enact consciously or unconsciously oppressive behaviors leading to an internalization inconsistent to what students perceive a scientist to be (Reynolds and Pope 1991). In other words, perhaps identity is better conceived as a general construct in which beliefs about identity with components of personal identity and perceived external identities.

Building upon these views of what identity is and does, we propose a conceptualization of identity that draws upon both the internalized view of self and the perceived view of external others regarding one’s science identity. The approach builds upon prior work of Hazari et al. (2010), but it was adapted for middle school and early high school. The primary goal was to understand the relationship between internal and perceived external elements of science identity and the independence from other attitudinal measures such as interest or competency beliefs, which others have conceptualized as part of identity.

This question of construct content can be examined from multiple perspectives. The current study focuses on two perspectives that are most effectively conducted through quantitative research: psychometric coherence (i.e., an individual differences construct is meaningful if its components cohere internally and discriminate against other constructs across individuals) and predictive validity (i.e., components form a coherent construct if each of the components does similar work above other attitudinal constructs for the individual). In particular for predictive validity, the second research question examines whether the different components of science identity each underlie preferences towards and actual participation in optional learning experiences.

It is possible that such quantitative measures and analysis obscure meaningful variation by context and subgroup, but such quantitative investigations do highlight patterns that hold across a broader set of learners and contexts. Specifically looking at broad patterns in a particular population reveals whether youth participation in optional science experiences at this age (and in their US urban context) is only determined by science identity or whether there are important regular interactions of science identity with patriarchal and racist values in science to hinder participation in science.

## Research questions

1. What are the components of science identity for students at this age that are distinct from other attitudinal constructs?
2. To what extent does science identity predict student’s choices overall and separately by gender and race/ethnicity?

## Methods

### Participants

Our sample is a subset of the ALES15 dataset (Activated Learning Enables Success 2015). This data was collected by a research team from the Activation Lab (activationlab.org) in a diverse range of public urban schools from two different regions in the USA with approval from the University of Pittsburgh and University of California-Berkeley Institutional Review Boards. The full dataset is longitudinal and includes a wide range of demographic, attitudinal, and experience measures, and is available upon request by contacting the Activation Lab team. The current study uses the subset of schools that participated in both pre and post data collection points reported in this study. The current analyses focus on the science identity scale, which has not been reported elsewhere.

The schools in this study were chosen to represent different historical emphases on STEM and different distributions of ethnicities. Data was collected from recruited 23 seventh grade, and 32 ninth grade classes from 19 public schools, also with widely varying demographics (minoritized groups in science, 23–99%; free/reduced lunch, 26–84%). Table 1 presents the overall demographic characteristics of each group. Overall, the sample is similar to US urban middle school students on key demographic distributions relevant to science education (e.g., sex and race/ethnicity) (Archer et al. 2012, Brown 2004; Oakes 1990), except for a slight over-representation of African Americans and under-representation of Hispanic/Latino and Asians (Kena et al. 2014): 50% White, 25% Hispanic/Latino, 16% African American, and 5% Asian.

**Table 1:** Participant age (in years), sex, and ethnicity information across grades

Grade	Age		% Female	Race/ethnicity				
	M	SD		% White	% Black	% Asian	% Latinx	% Other
7	13.5	0.6	51	56	34	9	12	12
9	15.4	0.6	50	49	41	10	11	11

Percentages add to more than 100% due to multi-ethnic identities

Sample sizes varied across measures due to student absence across data collection points. The primary sample of this study consisted of 1322 students. The percentage of missing item data for all the scales employed had a mean of 0.2% and no higher than 4.8% for any item. We therefore did not use data imputation methods since those are typically recommended for datasets with an average of 4% to 15% missing data (Newman, 2009). Instead, missing items were dropped from the computation of mean scores, and students simply needed to have at least half the items on a scale for a mean to be computed.

### Measures

Four types of constructs were assessed via surveys: (1) *Science identity*; (2) three other forms of science attitudinal factors to test discriminant validity; (3) two measures of optional science learning experiences to test predictive validity; and (4) multiple demographic measures.

#### Science identity

The *science identity* scale was designed to reveal the components of students’ endorsement of a science identity. The scale was adapted from Aschbacher et al. (2010) and Shanahan (2009) and designed in particular to test whether and which external components of science identity cohere with internal components as a construct: (1) *perceived personal science identity*, where students with high science identity would see themselves as being the kind of person who is associated with science, and (2) *perceived recognized science identity*, where they perceive that influential others (friends, family, and teachers) see them in this way (with one item per each of the three influential others). Ratings were given on a four-point Likert scale (4 = YES!, 3 = yes, 2 = no, 1 = NO!).

Psychometric properties of the *science identity* items (item means, standard deviations, and EFA statistics) are presented in Table 2. If treated as a coherent scale, the reliability is high (Cronbach’s alpha = .84, Polychoric alpha, which does not assume the Likert scale is an interval scale, = .88). The sample was split at random to create two independent groups to conduct the exploratory and confirmatory factor analyses (Browne and Cudeck 1993; Robida 2013).

**Table 2:** Mean, SD, and EFA factor loadings of the science identity scale

Survey items	Mean	SD	Factor loading
I am a science person	2.3	0.9	0.66
My family sees me as a science person	2.2	0.9	0.85
My friends see me as a science person	2.0	0.8	0.87
My teachers see me as a science person	2.3	0.9	0.63
Total	2.2	0.7	

An exploratory factor analysis (EFA) was conducted on the first random dataset subset and all items loaded into a single factor with acceptable loadings; see the “Analyses” section for more information on the EFA technique. That is, the external items did not separate from the internal item, nor did some of the external items separate from each other. A confirmatory factor analysis (CFA) on the remaining data produced satisfactory levels on all three fit statistics: (1) CFI = 0.99: the comparative fit index (ranging 0 to 1) tests how well the data fits the hypothesized unidimensional scale. Values of 0.95 or above are considered satisfactory; (2) TLI = 0.98: the Tucker-Lewis index (ranging 0 to 1) represents the extent to which the hypothesized model produces a better fit than a null model in which none of the items are assumed to be related to one another. Values of 0.95 or more are considered satisfactory; (3) RMSEA = 0.059: the root mean square error of approximation index (ranging 0 to 1) determines how well our model reproduces the data. Values of 0.06 or less are considered satisfactory (Costello and Osborne 2005). In sum, internal and all three external identity items cohere strongly as a single scale construct.

Finally, differential item functioning (DIF) analyses were conducted by gender, ethnicity, and age to test for measurement bias or differential functioning by subgroup. For example, it is possible that teacher perceptions are less meaningful in defining identity to older or minority students. Without this test, it would not be meaningful to make comparisons across those subgroups groups (Gregorich 2006). Importantly, we did not find any differential functioning by gender, race/ethnicity, or age on any of the identity items.

#### Attitudes towards science

Attitudes towards science can include ideas, values, beliefs, and perceptions regarding the general enterprise of science, school science, or another context where students interact with scientific knowledge and ideas (Gardner 1975). This study focused upon three commonly implicated constructs in science identity and previously found to be predictors of student choices. For information about the theoretical foundation, development, reliability, and validity of the scales, see (Dorph et al. 2016); in each case, scales were constructed using items that showed adequate psychometric properties for the sample studied.

1. *Fascination*—Fascination in science refers to interest and positive affect towards science, curiosity about the natural world, and goals of acquiring and mastering scientific skills and ideas. The scale (*α* = 0.83) was computed as a mean across the five items, each involving a four-point Likert scale (e.g. “I need to know how objects work.” 4 = YES!, 3 = yes, 2 = no, 1 = NO!).
2. *Values*—Values refers to the importance placed on knowing and being able to do science because of its usefulness in meeting personal goals (e.g., fixing a problem at home) and its utility to society (e.g., solving environmental problems). The scale (*α* = 0.73) was computed as a mean across three items, each involving a four-point Likert scale (e.g. “Knowing science helps me understand how the world works” 4 = YES!, 3 = yes, 2 = no, 1 = NO!).
3. *Competency beliefs—*Competency beliefs are the learner’s beliefs about their ability to successfully participate in diverse science learning situations as well as their beliefs about having the core skills of science to have a good performance in specific activities. The scale (*α* = 0.63) was computed as a mean across four items, each involving a four-point Likert scale (e.g. “ I can do the science activities I get in class” 4 = YES!, 3 = yes, 2 = no, 1 = NO!).

#### Choice preferences

Choice preferences for optional science learning experiences were measured as a mean of ten items (*α* = 0.85) on a Likert scale. These items ask about students preferences to participate in the future in common optional learning experiences involving science at home, at school, or in other locations (e.g. “I would like to attend a science camp next summer” 4 = YES!, 3 = yes, 2 = no, 1 = NO!). The choices ranged from situations that could happen in the immediate future to choices about preferences for the next year.

#### Science experiences

To complement the measure of student preferences, students were later asked what actual optional science learning experiences they had experienced in the intervening time since the initial *science identity* and other attitudinal measures were collected. The 12 items measured a range of recent experiences that students had recently had, many of which were STEM-related optional experiences. All items were measured on a four-point Likert regarding the amount of exposure to the experience (4 = many days, 3 = a few days, 2 = 1 day, 1 = never). These recent experiences were conceptually grouped by location (related to school or at home), based on prior work showing that relative amounts of experiences tended to group this way and had different effects on learners(Liu and Schunn 2018), and psychometric analyses. The resulting scales were the following:

1. *Formal recent science experiences* during the school year—Measured as the mean across seven items (*α* = 0.72) (e.g., “I did an extra-credit research project for science class”). Formal experiences were defined as optional science learning experiences that were school-related (i.e., happened in school after class hours or related to science class) but were not just regular homework activities.
2. *Informal recent science experiences* at home during the school year—Measured as the mean of five items (*α* = 0.77). Informal experiences refer to those experiences related to science that were not closely connected to formal curriculum and where students were free to explore the topics at their own pace (e.g., “I read books about science or science fiction”).

#### Demographics

Participants provided basic demographic information, from which variables were derived for sex, age, and race/ethnicity. Students were asked to select among four different gender identities (e.g., boy, girl, trans, non-binary); this study kept only the students that identified as boy and girl given the very low rate of the other two categories. Students were asked to select among six different racial/ethnicity categories with which they identified and were allowed to choose more than one. From this ethnicity data, a binary variable called *Minoritized Students* was created, with a 0 for students who selected only White or Asian, and 1 for students belonging to racial/ethnic groups underrepresented in science.

### Data collection procedure

Students completed all but one of the surveys early in the fall semester on paper during one class period as a single packet distributed by members of the research team. The administration procedure was consistent across schools. The demographic questions were given last in the packet to avoid the effect of stereotype threat on attitudinal survey responses (Steele and Aronson 1995). Students separately completed the survey regarding recent academic year science experiences (informal and formal) early in the spring semester.

### Analyses

Analyses were conducted and reported in the following order corresponding to the two research questions:

1. To understand whether *science identity* is different from other attitudinal constructs (discriminant validity), an exploratory factor analysis (EFA) was conducted using all the items from the four attitudinal scales. EFA is a statistical technique that uncovers how survey items should be grouped into empirically determined clusters according to similar response patterns. Each resulting group is thought to measure a different theoretical construct. In this study, the EFA tests whether the identity items are measuring a construct that is separable from the other three attitudinal constructs. The factor analysis was conducted using a promax rotation that allows the underlying factors to be correlated, which is almost always the case for attitudinal variables.
2. A multiple regression model was applied to the data to test whether science identity predicted subsequent student experience outcomes (predictive validity) and whether perceived personal and perceived external science identities serve different functions when predicting these outcomes. The model included other motivation constructs as possible predictors to establish the relative strength of identity in predicting student choices (i.e., is identity particularly important for predicting participation). The model also included demographic variables to show that it was identity per se rather than correlated demographic factors that predicted participation.
3. A more complex moderation analysis was conducted to understand whether there were differential relationships between science identity and subsequent science experiences across gender and ethnicity. A moderation analysis is a variant of the multiple regression in which interaction terms are added and tested for statistical significance. Moderation refers to when the relationship between two variables depends on a third variable (e.g., science identity may be highly predictive of science experiences for girls but not for boys). For statistical power reasons, the ethnicity analysis focused on White and Black students.

## Results

### Discriminant validity: is *science identity* a separate attitudinal construct for students in seventh and ninth grade?

The EFA applied to all the survey items from the science identity and attitudinal constructs returned a four-factor solution (four groups that are correlated with one another but are nonetheless distinctly different) in which perceived personal *science identity* and perceived recognized *science identity* components closely cohered. *Science identity* cleanly separated from the other three attitudinal measures without significant cross-loading (no item is loading in multiple factors; see Table 3). In fact, the cross-loadings of the other attitudinal items on the science identity factor were almost all below 0.1 (Costello and Osborne 2005). The same clean separation occurred with factor analyses conducted separately within each grade, sex, and race/ethnicity (see Additional file 1). These results suggest that in the middle school and early high school grades:

1. *Science identity* is psychometrically distinct from the other science attitudinal measures often attributed to identity.
2. Perceived personal *science identity* and perceived recognized *science identity* cohere strongly into one overall identity construct.

**Table 3:**
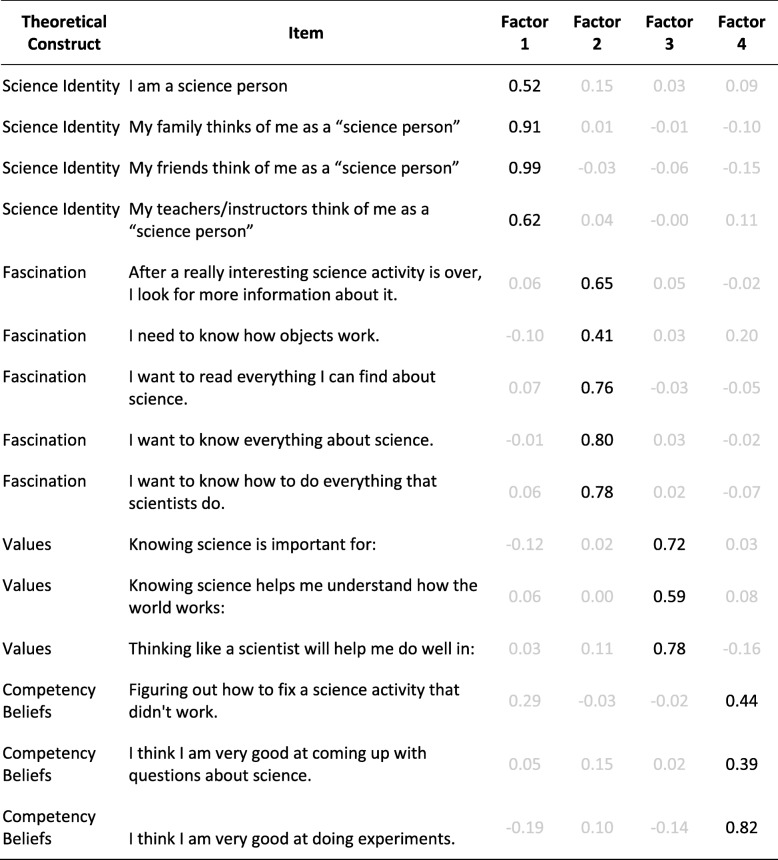
Exploratory factor analysis loadings for science identity, fascination, values, and competency belief survey items. Loadings below 3 are shown in gray font

### Consequential validity: differential relationships of *science identity* to participation on optional science experiences

To assess whether science identity serves as a critical attitudinal construct (i.e., predicts important learning behaviors), multiple regression tests examined whether it uniquely predicted participation in out-of-school science experiences above and beyond established other science attitudinal measures like fascination or competency beliefs. Follow-up analyses tested for similar contributions from each of the internal and external identity components.

Table 4 presents the means, standard deviations, and inter-correlations for each of the predictor and outcome measures. The standard deviation of each of the predictors is sufficiently large that each measure has sufficient variability to serve as an important predictor, and the inter-correlations among the predictors is sufficiently low that there should not be collinearity problems given the size of the dataset (except for overall identity against its two components). Mean and SD were also calculated within subgroups by gender and race/ethnicity to make sure there were no ceiling/floor effects or restricted range issues within a subgroup. Interestingly, the mean differences for *science identity* showed relatively small effects although statistically significant and in the expected direction by gender (*η*^2^ = 0.015, *p* = 0.001) and by race/ethnicity (*η*^2^ = 0.008, *p* = 0.015).

**Table 4:** Means, SD, and Pearson correlation coefficients among the attitudinal predictors and the optional science learning measures

	Mean	SD	Science identity	Perceived personal	Perceived external	Fascination	Values	Competency beliefs	Choice preferences	Formal science experiences
Identity	2.1	0.7								
Perceived Personal	2.1	0.9	0.81							
Perceived External	2.1	0.7	0.97	0.65						
Fascination	2.5	0.6	0.54	0.48	0.51					
Values	2.7	0.6	0.48	0.42	0.45	0.52				
Competency beliefs	2.9	0.5	0.47	0.46	0.42	0.50	0.44			
Choice preferences	2.4	0.6	0.44	0.40	0.41	0.48	0.38	0.35		
Formal science experiences	1.9	0.7	0.23	0.21	0.21	0.20	0.20	0.18	0.32	
Informal science experiences	2.3	0.7	0.30	0.27	0.28	0.32	0.26	0.23	0.61	0.35

The multiple regression results are shown in Table 5. Across all three measures of participation in optional science learning experiences, *science identity* not only significantly predicted participation (preferred and actual of both types), it was always one of the stronger predictors, and for informal, it was the strongest predictor (Table 5). The relatively weaker predictions for formal experiences may be explained by relative levels of access (e.g., whether the school provided access and whether the student’s family attended museums in general), as suggested by the lower *R*^2^ for the overall model using attitudinal variables. Note that although the predictors were correlated with one another, there were no problems of separating the individual contributions of each predictor in any of the regression models as indicated by relatively low variable inflation factors (VIF). These results suggest:

**Table 5:** Multiple regressions of attitudinal factors predicting different out-of-school science experiences controlling for sex and race/ethnicity, along with overall model *R*^2^ and the largest VIF value in each model

	Choice preferences		Formal science experiences		Informal science experiences	
	*β*	*p*	*β*	*p*	*β*	*p*
Science identity	0.22	< .001	0.15	< .001	0.27	< .001
Fascination	0.27	< .001	0.18	< .001	0.11	0.008
Values	0.09	0.02	0.09	0.04	0.09	0.02
Competency beliefs	0.08	0.04	0.04	0.37	0.10	0.008
*R*^2^ total	0.28		0.13		0.21	
Max VIF	1.75		1.74		1.74	

1. *Science identity* overall is a strong predictor of students’ science-related choices

2. *Science identity* behaves separately from other attitudinal factors and has a unique contribution to our understanding of students’ choices.

Table 6 shows the results of the follow-up multiple regressions with separate perceived personal and perceived recognized scores (along with other attitudinal covariates) in predicting the three measures of participation in science experiences. Most importantly, the standardized beta loadings for personal and recognized *science identity* are of similar size, arguing against a mediated relationship of personal *science identity* through recognized *science identity*. Moreover, when these analyses were repeated on subsets of the data by sex, race/ethnicity, and their intersection (e.g., black women vs. white women), the same pattern of roughly equal contributions of personal and recognized identities were observed (see Additional file 1). Overall, these results support a coherent personal/recognized *science identity* across gender and race/ethnicity subgroups.

**Table 6:** Multiple regressions comparing perceived personal vs. perceived external science identity factors. All models include fascination, values, and competency beliefs as covariates

Science identity component	Choice preferences		Formal science experiences		Informal science experiences	
	*β*	*p*	*β*	*p*	*β*	*p*
Perceived personal	0.10	< .001	0.06	0.090	0.20	< .001
Perceived external	0.14	0.002	0.10	< .001	0.11	0.001
*R* ^2^	0.28		0.14		0.23	
Max VIF	1.97		1.97		1.97	

Finally, moderation models formally tested whether there were differential relationships of science identity with participation in optional science experiences by gender or race/ethnicity. Overall, interactions with gender were commonly observed and generally consistent across a measure of science identity (see Table 7). Interactions with race were rarely observed, once at the level of perceived personal and once at the level of perceived external.

**Table 7:** Statistical significance of interactions between identity and gender or race in predicting optionally preferred and actual science experiences across science identity types

	Choice preferences			Formal experiences			Informal experiences		
	Internal	External	Overall	Internal	External	Overall	Internal	External	Overall
Identity × gender	**	**	**	*		**	**	**	**
Identity × race				*				*	

*p < 0.05;**p < 0.001

**Fig. 1:**
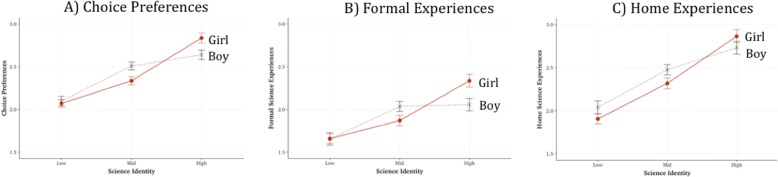
Estimated marginal means (with SE bars) of **a** choice preferences, **b** formal science experiences, and **c** home science experiences, predicted by science identity (low, moderate, and high levels) and gender

Given the more consistent pattern of results by gender across identity measures, detailed descriptive statistics are provided for just those interaction effects. Students were binned into three levels of overall *science identity* (*binned into equal frequency bins, with 33% of responses in each bin*). The interactions are shown in Fig. 1a–c. All three figures show a similar pattern: at moderate levels of science identity, boys participated more often than girls in optional experiences, whereas at high levels of science identity, the opposite pattern is observed.

## Discussion

Understanding student’s identity in science is important as it is an important driver of choice in their present and in their future (Barton et al. 2013; Crowley et al. 2015; Hazari et al. 2010). Furthermore, identity is a multicomponent construct through which people internalize experiences, their context, see themselves as members of social groups, and intersect with their personal characteristic (e.g., gender and race). This section revisits each of the primary research questions to discuss the theoretical and practical implications of our findings and how context relates to science identity.

### Which attitudinal aspects cohere within science identity?

Many researchers have strongly connected science identity with attitudinal constructs like fascination, values, and competency beliefs (Barton et al. 2013; Barton and Tan 2010; Brotman and Moore 2008; Hazari et al. 2010; Trujillo and Tanner 2014), and some measures of identity occasionally even include items which appear more similar to items typically found in measures of these other constructs (Crowley et al. 2015; Eccles et al. 2015; Trujillo and Tanner 2014). Critically, the four-factor solution from the EFA analyses revealed that *science identity* was psychometrically distinct from the other science attitudinal measures, contrary to the prior conceptualizations of identity as a super-ordinate construct. Moreover, perceived personal *science identity* and the three different perceived recognized *science identity* elements loaded into a single identity factor. Although some research has argued through qualitative research methods that perceived personal and perceived recognized factors separate specifically when looking at different ethnic/racial groups (Barton et al. 2013; Rosa and Mensah 2016), we did not find this separation—in this urban context with middle-schoolers and early high-schoolers, we observed a consistent loading into a single factor across racial/ethnic groups, gender, and grade. It is possible that, as students grow older, these factors separate, especially in highly masculine science fields like physics and engineering (Hazari et al. 2013) due to lack of external support and need for a lot of internal resilience. Such possible effects highlight the importance of the context and its likely role on student’s internalization of science identity. But, is also important to understand that at least at this age, perceived personal and perceived external factors are closely related and these appeared to contribute to students’ overall science identities. Furthermore, science identity was the only attitudinal construct that has a strong component influenced directly by the student’s context (perceived recognized *science identity).* Interestingly, at this age, there was no evidence of large gaps in students’ science identity by any of the examined demographic subgroups or their interaction. It may be that contextual factors commonly associated with students’ internalization of negative stereotypes or barriers around gender and race/ethnicity have not become salient at this age in this middle and early high school context.

### Is science identity predictive of student choices?

Science identity overall was either comparable or a stronger predictor of out-of-school science experiences when compared to other attitudes towards science already associated with making such choices (Dorph et al. 2016; Lin and Schunn 2016). This is an important finding in terms of increasing our understanding of the factors that support students’ pathways towards science careers.

Furthermore, although the factor analyses established relatively high covariance of perceived personal and perceived recognized aspects of science identity, these analyses on their own did not establish that both personal and recognized aspects serve similar functions such as the decisions to participate in optional science learning experiences. It was still possible based on those analyses alone, as others have argued (Hazari et al. 2010; Rosa and Mensah 2016), that perceived external identity influences personal identity, and then personal identity drives participation. However, the follow-up regression analyses revealed that both perceived personal and perceived recognized identities were similarly important in predicting participation in out-of-school science experiences. Moreover, the importance of both aspects in predicting choices were similar across gender, race/ethnicity, and grade. In terms of theory, these results suggest that, at this developmental stage, perceived personal and perceived recognized identities are so closely related that can be measured and understood as a single functional construct. In other words, identity emerges from both views of self and perceptions of how others view the individual, and both elements shape the choices learners wish to make as well as the choices they actually make. More specifically, these perceptions are shaped by students’ context and is highlighted by the coherent and central role of perceived recognized *science identity*. This pattern is not only consistent with current understandings of how systemic disadvantages can affect science pathways, but, it may also suggest opportunities for intervention: influential others could be used to change activity patterns and change self-perceptions of identity, leading to further changes in activity.

Finally, although identity mattered (and both aspects of identity mattered) to guide participation in optional science for all subgroups, they did so to differ extents across gender subgroups: for girls, science identity played a greater role in shaping participation in science. Unfortunately, girls also had somewhat lower overall science identities. That is, the groups most dependent upon science identity for participation also had lower overall identities. This pattern partially explains the gap in participation by gender. However, further research is required to understand the mechanism underlying the particular patterns that were observed. Most saliently, why was participation lower in girls at moderate levels but greater at high levels of science identity?

### Generalizability of the patterns and limitations

This study purposely sampled students from diverse public urban schools in one region of the USA to understand the nature of science identity and its relationship to personal characteristics (e.g., race and gender) as well as optional science experiences. While the proportion of minoritized youth within the study sample was an overall match to base-rates in US urban public schools, the distribution by more specific subgroups was not (Aschbasher and Roth 2010). Further, a much larger sample is required to produce truly representative data for urban students in the USA, including other regions as well as private, charter, and home-schooled students. Furthermore, a much larger sample would also allow us to draw inferences in other important demographic groups like Latinx students.

Another possible limitation is regarding our choice of grouping of seventh and ninth graders together, as arguably the students could be in very different developmental processes and therefore may internalize science identity differently. However, at both seventh and ninth grade, students experience science for one period per day, and ninth grade is still before students enroll in more advanced science/Advanced Placement (AP) type courses. Additionally, we conducted a variety of validity checks to ensure that the patterns and measures within each of the two grades were similar: (1) a DIF analysis by grade showing measurement invariance, (2) regression analysis separately by grade finding similar patterns, and (3) the science identity mean was not statistically different between groups.

Finally, it is important to note that this sample was collected within a formal context. It is possible that patterns of behavior and influence of science identity could look different within informal settings.

## Conclusions

This study found results consistent with many identity theorists’ conceptions of identity as a multicomponent construct (Gee 2000). The novel contribution to the science identity field highlights the specific multi-component ways in which students endorse science identity in middle school and early high school. At this age in this particular context, across gender and race/ethnicity subgroups, science identity appears to be a cohesive construct conformed by perceived personal and perceived recognized internalizations of science identity. This result is important because it highlights the importance of students’ context on their construction of their science identity. It also creates opportunities for interventions that can have an impact on overall science identity—via interventions focused on changing perceptions others convey about a students’ science identity. Further, although at this age and particular context, there were relatively small differences by gender, race/ethnicity, and age, there was an important finding that science identity has a complex differential function in supporting student’s optional science choices by gender. Thus, at this age, developing a strong science identity is especially critical for girls. These findings highlight the importance of looking beyond mean differences across subgroups. These results also demonstrate important findings that quantitative methods can produce to deepen understanding of how attitudinal and identity constructs can have differential effects across subgroups.

## Additional file

Additional file 1: Factor analyses conducted separately within each grade, sex, and race/ethnicity. (DOCX 19 kb)
