# Supplementary material for: The nature of science identity and its role as the driver of student choices
Source: Int J STEM Educ. 2018 Nov 30;5(1):48. doi: 10.1186/s40594-018-0140-5 (PMC6310435; doi:10.1186/s40594-018-0140-5)
Supplement: Supplementary file 1 — Factor analyses conducted separately within each grade, sex, and race/ethnicity. (DOCX 19 kb) [file 40594_2018_140_MOESM1_ESM.docx]

# Factor analyses conducted separately within each grade, sex, and race/ethnicity

| **Factor Analysis for Girls** | | | | |
| --- | --- | --- | --- | --- |
|  | **Factor 1** | **Factor 2** | **Factor 3** | **Factor 4** |
| T40_F05 | 0.58 | -0.01 | 0.10 | 0.09 |
| T40_F06 | 0.40 | -0.02 | 0.10 | 0.04 |
| T40_F07 | 0.84 | 0.06 | -0.03 | -0.12 |
| T40_F08 | 0.83 | 0.03 | 0.02 | -0.05 |
| T40_F09 | 0.78 | 0.08 | -0.02 | -0.03 |
| T40_V02 | 0.03 | -0.14 | 0.77 | -0.07 |
| T40_V03 | 0.02 | 0.07 | 0.56 | 0.07 |
| T40_V05 | 0.05 | 0.02 | 0.73 | -0.07 |
| T40_CB01 | -0.10 | 0.06 | 0.06 | -0.18 |
| T40_CB06 | -0.10 | 0.20 | 0.04 | 0.60 |
| T40_CB09 | -0.03 | 0.06 | 0.20 | 0.36 |
| T40_CB10 | -0.09 | -0.03 | -0.02 | 0.61 |
| T40_SCID01 | 0.21 | 0.53 | 0.04 | 0.09 |
| T40_SCID10 | -0.01 | 0.91 | 0.00 | -0.06 |
| T40_SCID11 | 0.01 | 1.00 | -0.12 | -0.13 |
| T40_SCID12 | 0.07 | 0.58 | -0.04 | 0.14 |
| *Cronbach's α* | *0.84* | *0.86* | *0.71* | *0.25* |

| **Factor Analysis for Boys** | | | | |
| --- | --- | --- | --- | --- |
|  | **Factor 1** | **Factor 2** | **Factor 3** | **Factor 4** |
| T40_F05 | 0.67 | 0.03 | -0.01 | 0.02 |
| T40_F06 | 0.38 | -0.09 | 0.06 | 0.19 |
| T40_F07 | 0.81 | 0.08 | -0.09 | -0.03 |
| T40_F08 | 0.76 | 0.04 | -0.02 | -0.01 |
| T40_F09 | 0.77 | 0.01 | 0.06 | -0.11 |
| T40_V02 | -0.09 | -0.02 | 0.77 | -0.00 |
| T40_V03 | 0.03 | 0.02 | 0.58 | 0.06 |
| T40_V05 | 0.09 | 0.02 | 0.76 | -0.14 |
| T40_CB01 | -0.01 | 0.11 | -0.03 | -0.32 |
| T40_CB06 | -0.09 | 0.32 | 0.05 | 0.40 |
| T40_CB09 | 0.22 | 0.11 | 0.05 | 0.30 |
| T40_CB10 | -0.05 | 0.03 | -0.15 | 0.88 |
| T40_SCID01 | 0.04 | 0.55 | 0.06 | 0.11 |
| T40_SCID10 | 0.06 | 0.83 | -0.02 | -0.12 |
| T40_SCID11 | -0.04 | 0.96 | -0.01 | -0.14 |
| T40_SCID12 | 0.07 | 0.67 | -0.01 | -0.01 |
| *Cronbach's α* | *0.82* | *0.84* | *0.73* | *0.20* |

| **Factor Analysis for White students** | | | | |
| --- | --- | --- | --- | --- |
|  | **Factor 1** | **Factor 2** | **Factor 3** | **Factor 4** |
| T40_F05 | 0.03 | 0.56 | 0.10 | 0.05 |
| T40_F06 | 0.03 | 0.41 | 0.05 | 0.01 |
| T40_F07 | 0.08 | 0.80 | -0.02 | -0.09 |
| T40_F08 | 0.07 | 0.78 | -0.00 | -0.03 |
| T40_F09 | 0.05 | 0.80 | -0.01 | -0.05 |
| T40_V02 | -0.14 | -0.07 | 0.84 | -0.01 |
| T40_V03 | -0.02 | 0.16 | 0.52 | 0.08 |
| T40_V05 | 0.00 | 0.12 | 0.69 | -0.11 |
| T40_CB01 | 0.13 | -0.15 | 0.09 | -0.24 |
| T40_CB06 | 0.26 | -0.09 | 0.05 | 0.55 |
| T40_CB09 | 0.15 | 0.08 | 0.17 | 0.29 |
| T40_CB10 | 0.01 | -0.16 | 0.04 | 0.72 |
| T40_SCID01 | 0.56 | 0.14 | 0.05 | 0.11 |
| T40_SCID10 | 0.91 | 0.02 | -0.02 | -0.09 |
| T40_SCID11 | 0.96 | 0.01 | -0.08 | -0.10 |
| T40_SCID12 | 0.69 | 0.09 | -0.12 | 0.03 |
| *Cronbach's α* | *0.86* | *0.83* | *0.72* | *0.26* |

| **Factor Analysis for Minority Students** | | | | |
| --- | --- | --- | --- | --- |
|  | **Factor 1** | **Factor 2** | **Factor 3** | **Factor 4** |
| T40_F05 | 0.06 | 0.63 | -0.03 | 0.07 |
| T40_F06 | -0.11 | 0.39 | 0.06 | 0.19 |
| T40_F07 | 0.05 | 0.82 | -0.06 | -0.06 |
| T40_F08 | 0.00 | 0.84 | -0.01 | -0.04 |
| T40_F09 | 0.10 | 0.73 | 0.04 | -0.10 |
| T40_V02 | -0.12 | -0.05 | 0.81 | -0.04 |
| T40_V03 | 0.10 | -0.01 | 0.59 | 0.02 |
| T40_V05 | 0.04 | 0.08 | 0.77 | -0.17 |
| T40_CB01 | 0.03 | -0.03 | -0.09 | -0.21 |
| T40_CB06 | 0.35 | -0.12 | 0.11 | 0.31 |
| T40_CB09 | 0.01 | 0.10 | 0.09 | 0.35 |
| T40_CB10 | -0.03 | -0.06 | -0.21 | 0.91 |
| T40_SCID01 | 0.55 | 0.13 | 0.03 | 0.10 |
| T40_SCID10 | 0.84 | 0.02 | -0.04 | -0.03 |
| T40_SCID11 | 0.98 | -0.03 | -0.09 | -0.13 |
| T40_SCID12 | 0.62 | 0.11 | 0.04 | -0.00 |
| *Cronbach's α* | *0.84* | *0.83* | *0.72* | *-0.06* |

| **Factor Analysis for 7^th^ Grade** | | | | | |
| --- | --- | --- | --- | --- | --- |
|  | **Factor 1** | **Factor 2** | **Factor 3** | **Factor 4** | **Factor 5** |
| T40_F05 | 0.65 | 0.04 | 0.00 | -0.05 | 0.16 |
| T40_F06 | 0.43 | -0.08 | 0.03 | 0.10 | 0.03 |
| T40_F07 | 0.87 | 0.13 | -0.15 | -0.19 | 0.24 |
| T40_F08 | 0.79 | -0.05 | 0.08 | 0.08 | -0.24 |
| T40_F09 | 0.75 | -0.01 | 0.05 | -0.02 | -0.10 |
| T40_V02 | -0.03 | -0.07 | 0.73 | 0.01 | 0.10 |
| T40_V03 | -0.01 | 0.08 | 0.58 | 0.03 | 0.06 |
| T40_V05 | 0.05 | 0.04 | 0.79 | -0.12 | -0.02 |
| T40_CB01 | -0.01 | 0.02 | -0.06 | -0.09 | -0.23 |
| T40_CB06 | -0.05 | 0.16 | 0.01 | 0.60 | 0.04 |
| T40_CB09 | 0.12 | 0.09 | -0.02 | 0.45 | 0.07 |
| T40_CB10 | -0.03 | -0.12 | -0.04 | 0.69 | 0.07 |
| T40_SCID01 | 0.18 | 0.46 | 0.03 | 0.20 | -0.21 |
| T40_SCID10 | -0.04 | 0.94 | 0.00 | -0.07 | -0.05 |
| T40_SCID11 | 0.00 | 0.93 | -0.00 | -0.13 | -0.00 |
| T40_SCID12 | -0.02 | 0.64 | 0.01 | 0.08 | 0.10 |
| *Cronbach's α* | *0.82* | *0.85* | *0.75* | *0.64* |  |

| **Factor Analysis for 9^th^ Grade** | | | | |
| --- | --- | --- | --- | --- |
|  | **Factor 1** | **Factor 2** | **Factor 3** | **Factor 4** |
| T40_F05 | 0.08 | 0.63 | 0.00 | 0.01 |
| T40_F06 | -0.02 | 0.25 | 0.20 | 0.25 |
| T40_F07 | -0.01 | 0.81 | -0.05 | -0.05 |
| T40_F08 | -0.05 | 0.90 | -0.01 | -0.04 |
| T40_F09 | 0.02 | 0.81 | -0.03 | -0.00 |
| T40_V02 | -0.13 | -0.11 | 0.80 | 0.04 |
| T40_V03 | 0.01 | 0.04 | 0.58 | 0.10 |
| T40_V05 | -0.05 | 0.12 | 0.72 | -0.13 |
| T40_CB01 | 0.09 | -0.00 | 0.09 | -0.15 |
| T40_CB06 | 0.39 | 0.01 | -0.00 | 0.26 |
| T40_CB09 | 0.10 | -0.01 | 0.16 | 0.36 |
| T40_CB10 | 0.03 | -0.03 | -0.01 | 0.82 |
| T40_SCID01 | 0.56 | 0.01 | 0.14 | 0.06 |
| T40_SCID10 | 0.87 | 0.01 | -0.06 | -0.02 |
| T40_SCID11 | 1.04 | -0.04 | -0.20 | -0.10 |
| T40_SCID12 | 0.77 | 0.05 | -0.06 | 0.04 |
| *Cronbach's α* | *0.84* | *0.85* | *0.71* | *0.35* |
